# Thermal Stereolithography of SiC-Loaded Acrylate Resins with Polymer-Derived Ceramic Infiltration

**DOI:** 10.1021/acsaenm.5c00054

**Published:** 2025-04-01

**Authors:** Evelyn Wang, Shruti Gupta, Joseph Fortenbaugh, Caillin J. Ryan, Christopher M. DeSalle, Jeffrey R. Shallenberger, Douglas E. Wolfe, Benjamin J. Lear, Michael A. Hickner

**Affiliations:** † Department of Material Science and Engineering, Pennsylvania State University, University Park, Pennsylvania 16801, United States; ‡ Department of Chemical Engineering and Materials Science, Michigan State University, East Lansing, Michigan 48824, United States; § Materials Research Institute, The Pennsylvania State University, University Park, Pennsylvania 16802, United States; ∥ Oak Ridge National Laboratory, Oak Ridge, Tennessee 37830, United States; ⊥ Department of Chemistry, Pennsylvania State University, University Park, Pennsylvania 16801, United States

**Keywords:** NIR thermal SLA, thermal polymerization, PDC composite, polymer-derived ceramic, mechanical properties

## Abstract

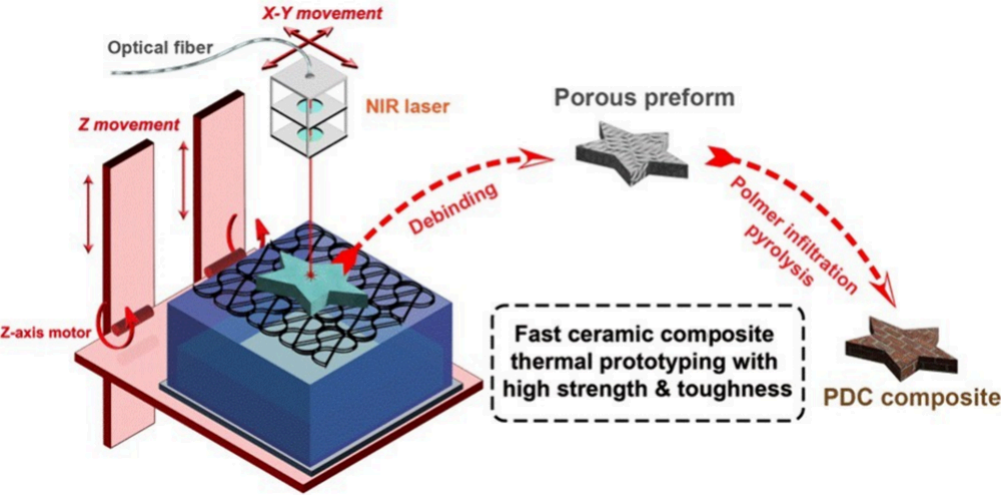

The implementation of stereolithography (SLA) for fabricating 3D-structured polymer-derived ceramics (PDCs) has greatly improved the resolution, manufacturing potential, and widespread capability to produce complicated component geometries in ceramic materials. However, different material systems impose challenges to the traditional UV SLA photo-cross-linking process due to a narrow window of material selection requirements—UV transparency, UV degradation resistance, the ability to support the photoinduced radical curing mechanism, and ambient shelf life stability. Herein, a near-infrared (NIR) thermal SLA printing technology is demonstrated on a composite thermally curable acrylate-based highly loaded resin to overcome current issues with UV light-driven SLA additive manufacturing of preceramic polymers (PCP). For this thermal SLA cross-linking method, a high-intensity NIR laser (λ = 808 nm) was used to generate localized thermal heating at the resin pool interface, which led to rapid, targeted thermal free-radical polymerization and solidification of the SiC particle-laden acrylate-based resin during laser scanning. Thermally cured printed parts were demonstrated using a gantry-based movement platform and a resin pool in a top-down laser scanning configuration. After printing, the green bodies were debinded, followed by polymer infiltration and pyrolysis (PIP) during postprocessing, which enhanced the mechanical strength of the pyrolyzed samples. This work demonstrated the fabrication of a reinforced PDC composite material with crystalline silicon carbide (SiC) fillers and an amorphous matrix made of silicon oxycarbide (SiOC) and silicon carbonitride (SiCN). The flexural strength of the NIR-printed samples reached 48 MPa with a fracture toughness of 4 MPa·m^1/2^.

## 1 Introduction

The low density, thermal resistance, and high-strength properties of silicon carbide (SiC) have attracted attention for aerospace, turbine, and electronic applications.^1^ However, as an abrasive material, SiC is difficult to machine into high-fidelity structures with complicated shapes. Therefore, new methods are needed for shaping SiC and related carbides, using novel processing techniques.^1^ As an emerging method for fabricating complex, 3D structures, additive manufacturing has been adapted to many materials, namely polymers, metals, composites, and ceramics.^2^ Polymer-derived ceramic (PDC) materials stand out as excellent candidates for additive manufacturing due to the potential to shape components in an easy-to-process polymeric form before ceramic conversion,^3^ which has been demonstrated in several examples.^4^

Preceramic polymers (PCPs) such as polysilazanes and polycarbosilanes are promising targets for 3D printing as their pyrolysis yields SiCN^5^ and SiOC^6^ materials with excellent mechanical properties and high ceramic yield. As demonstrated by Wang et al.,^7^ additive manufacturing methods such as SLA can be applied to the rapid fabrication of silicon oxycarbide (SiOC) 3D structures through free-radical cross-linking and solidification, including thiol–ene cross-linking of PCPs. Ceramic materials such as silicon carbonitride (SiCN),^5^ SiC,^8^ and PDC composites^9^ have been fabricated through stereolithography. Additionally, PCP resins can also be formulated with ceramic particles to improve the overall performance of the materials and increase the ceramic yield during processing.^11^

PCPs are usually processed thermally in conventional manufacturing, and these thermal processes have been adapted to suit various additive manufacturing methods. There are three major conventional 3D printing methods for PCPs: material extrusion, laser powder bed fusion, and sheet lamination. Direct ink writing (DIW) and fused filament fabrication (FFF) are examples of material extrusion-type printing. For DIW, heat is applied upon extrusion of the material from a nozzle to cross-link the PCP and solidify the printed structure. Kemp et al.^10^ designed a composite ink with boron nitride particles and PCP, which cures at elevated temperatures with the help of thermal initiators. Gorjan et al.^11^ proposed a printing method where PCP combined with plasticizer and ceramic fillers are used as the solid feedstock, which will melt upon heating, and the extruded filament will solidify upon cooling, mimicking typical polymer-based FFF. Selective laser curing (SLC) is an example of laser powder bed fusion, a process very similar to selective laser sintering (SLS) of polymers and metals. Friedel et al.^12^ showed that with constant recoating of the feed material, a CO_2_ laser can melt the PCP and demonstrated printing a 3D structure from a PCP powder bed composed of a mixture of PCP and SiC particles. Laminated object manufacturing (LOM) is an example of sheet lamination PCP printing. Sieber et al.^13^ demonstrated a LOM process for PCP processing, where paper sheets infiltrated with PCP are stacked together with hot pressing to fuse the structure.

There have been several previous reports on the SLA of PCPs.^3,14^ However, the majority of the work has emphasized UV-based SLA^15^ and digital light processing (DLP),^15^ since acrylate-based photoresins rapidly cure under UV light irradiation,^7^ which gives high-resolution and structural flexibility.^16^ A higher solid content of crystalline ceramic particles is desired in PCP formulations,^28^ as these fillers reduce the overall shrinkage during pyrolysis, preserve the structural integrity of the samples,^16^ increase ceramic yield,^4^ and strengthen the final parts.^9^ Thermal SLA, as proposed here, solves many issues encountered in UV-based photo-cross-linking SLA printing, where a wider selection of polymers and curing mechanisms are available for the printing, and the particle content in the printing ink can be greater than 20 wt % (upper limit of many UV-printing techniques),^9^ which ensures high crystallinity and high-density ceramic parts after pyrolysis. Thermal printing decreases the need for postprocessing of the samples, where the samples are strong enough to undergo a direct debinding or pyrolysis process. Additionally, the pot life of thermal resin can be longer than that of traditional UV resins,^1^ and no light inhibitors are needed. Due to the intrinsic limitations of UV printing,^8,17,18^ several studies have already been conducted on converting IR laser into UV light with up-conversion nanoparticles and IR initiators^19^ in SLA processing. These prior reports demonstrate the usefulness of NIR laser thermal curing over UV curing, including higher penetration depth and less structural damage.^20^

Laser-based technology has attracted much attention in materials processing, including processes such as stereolithography, cutting, localized heating, and probing.^21^ The earliest use of thermal laser-assisted 3D printing of polymers was in 1994,^21^ when a high-intensity 10.6 μm CO_2_ laser was used for 3D printing of a ring structure with silica-filled epoxy and polyester. High temperatures (200–280 °C) are often associated with CO_2_ laser excitation, which leads to burning and distortion of the printed structure.^22^ After the debut of additive manufacturing with IR laser excitation, researchers continued to tune IR laser-based processes in making well-defined 3D structures using powder bed fusion-based processing to avoid overheating the materials, controlling heat diffusion and object resolution, and monitoring the high-temperature process. Fu^23^ compared a visible 532 nm laser with a 10.6 μm CO_2_ laser, which was used in curing thermal-initiated resins, where it was discovered that both UV and IR wavelengths can cause structural damage by electronic and vibrational excitation, respectively. Fortenbaugh et al.^24^ extended the application of a 532 nm laser and applied the photothermal^25^ effect on silicone hydrosilylation using 100 nm gold nanoparticles. It was observed that light-absorbing fillers like gold nanoparticles promote a massive increase in the curing speed due to the light-to-heat conversion of the photothermally active particles. NIR laser excitation, with wavelengths in the range of 750 to 1300 nm,^26^ stands out as an excellent source of heat as it preserves the polymeric structures without significant chemical damage.^27^ More recent work also utilized a NIR laser in direct ink writing of optical silicones^28^ and a CO_2_ laser for SiC binder jetting,^12^ demonstrating the precision and printing capabilities of laser-based technology with different printing techniques.

This work reports a novel thermal SLA method for cross-linking highly loaded PCP resins. Thermally cured, acrylate-based resins with high SiC particle loadings (50 wt %) are demonstrated for various printed structures. The printed polymers were debinded and subjected to polymer infiltration and pyrolysis (PIP) to obtain excellent mechanical properties under low pyrolysis temperatures (800 °C).^25,26^ The thermal-curing SLA technique offers significant advantages due to its versatile resin composition and particle loading capabilities, allowing various fillers at high volume percentages, provided that the particles absorb sufficient IR light to cure the resin. Additionally, this technology supports a broad range of thermal curing reaction mechanisms, offering greater flexibility compared to UV-based photopolymerization techniques.

## 2 Experimental Section

### 2.1 Materials

**Figure 1:**
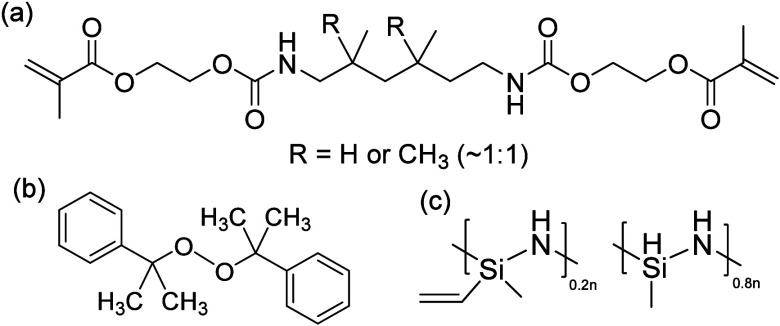
Chemical structures of (a) DUDMA, (b) dicumyl peroxide, (c) Durazane 1800.

All chemicals (Figure 1) were used as-received without further purification. Diurethane dimethacrylate (DUDMA), acetone (99.5%), and dicumyl peroxide (98%) were purchased from Sigma-Aldrich (St. Louis, MO). Durazane 1800 as the preceramic polymer (PCP) was supplied by Merck KGaA (Darmstadt, Germany), and silicon carbide particles (1 μm, β-phase, > 99.5%) were purchased from Beantown Chemical (Hudson, NH).

### 2.2 Sample Preparation

The preparation of the resin formulation consists of two steps. First, 100 g of DUDMA with an equal mass of SiC particles was transferred into a round-bottom flask, with 1 wt % dicumyl peroxide added as a thermal initiator. Then, 50 mL of acetone was added to the flask, and the mixture was subsequently stirred at 600 rpm for 10 h. Finally, all the solvent was removed with a rotary evaporator, which gave the resin composition (*DUDMA-SiC 50:50*) for subsequent printing. Durazane 1800 was premixed with 1 wt % dicumyl peroxide for the polymer infiltration process, as described in the following sections.

### 2.3 NIR Thermal SLA Printer

**Figure 2:**
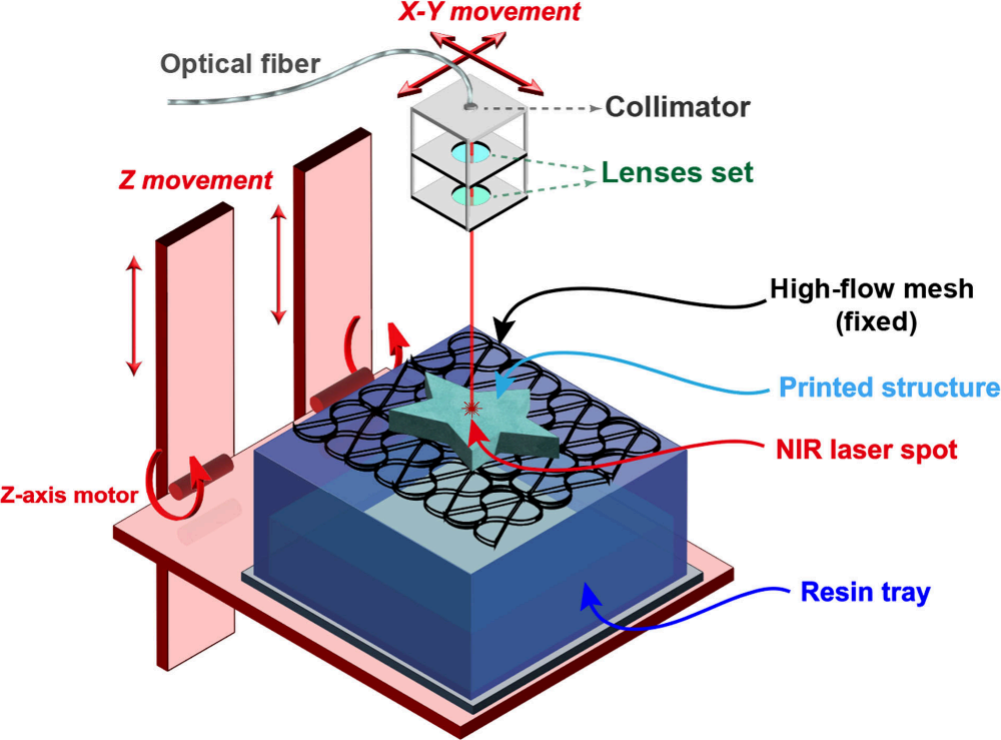
Thermal SLA printer schematic for thermal printing of the resin composition *DUDMA-SiC 50:50*.

For fabrication of the thermal SLA printer, all optics were purchased from Thorlabs Inc. (Newton, NJ), and a Lumics (Berlin, Germany) 808 nm fiber laser was used as the thermal laser source for printing (see equations (S1 and S2) for laser parameters). The printer consists of three parts–the NIR laser with an optical cage, the X-Y-Z gantry that controls the laser movement, and a stainless-steel baseplate mesh to support the printed structure, Figure 2.

For the NIR laser, an optical fiber was used to guide the laser generated from the 808 nm diode laser to the printing position. The advantage of using a fiber-coupled laser optic is that the laser module does not need to be attached to the moving print head, thus facilitating top-down SLA printing. The optic fiber was connected to a cage system, where the laser passed through a collimator and a set of lenses before focusing on the sample surface, where more details are illustrated in Figure S1.

For the printer and the X-Y-Z gantry movement stage, two motors control the x-y position of the laser to cure the resin at programmed locations. The *z*-axis movement added new layers to the printed structure, where a fixed stainless-steel mesh supported the printed structure while the resin tray was moved upward with the *z*-axis to immerse the sample in the resin pool further. The travel speed of the laser was set to be much faster (250 mm/s) than the printing speed (5 mm/s) to ensure it was not curing the empty area between the printing parts. A thermal camera (FLIR C3-X Compact Thermal Camera, Wilsonville, OR) was fixed onto the setup to monitor the printing temperature, where a temperature range of 140 to 160 °C was maintained to avoid both overcuring and undercuring without burning the resin (Video S1).

### 2.4 Green Body Formation

**Figure 3:**
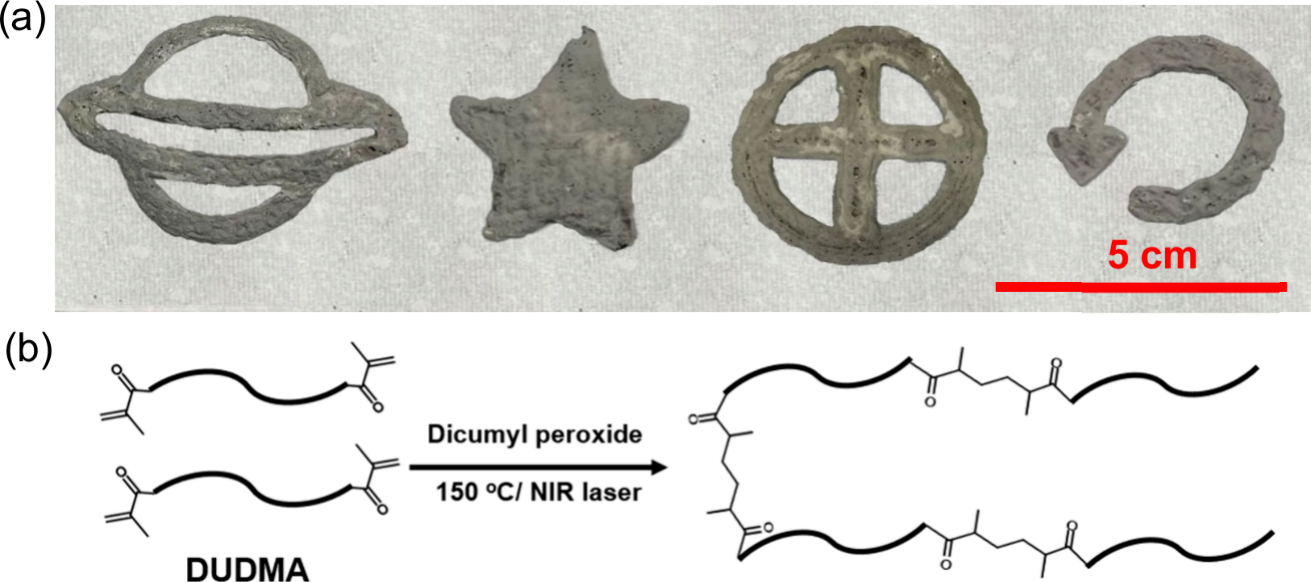
(a) Thermal SLA printed green body structures; (b) chemical cross-linking reaction during the thermal SLA printing.

Samples with a range of geometries were fabricated with the thermal SLA printer using the *DUDMA-SiC 50:50* resin, and the resulting single-layer green body structures are shown in the photograph in Figure 3(a) (samples after pyrolysis postprocessing are shown in Figure S2). A schematic of the thermal cross-linking is shown in Figure 3(b). To baseline chemical compositions and mechanical properties, oven-cured samples with the same resin composition were fabricated and processed in silicone molds. Oven curing of the samples was performed by heating the resin in a convection oven at 150 °C for 5 min, during which the resin was cured in a 2 cm × 5 cm bar-shaped specimen.

### 2.5 Polymer Infiltration and Pyrolysis

The first step in processing the as-printed green body sample is debinding, during which most of the organics that will not become ceramic are removed. The debinding process removes all the unpolymerized and polymerized acrylate resin from the samples to increase the porosity for PIP. Green body samples were heated in air at 0.4 °C/min to 500 °C in a muffle furnace and held for 1 h to remove all the polymeric species. The sample was then cooled to room temperature at a rate of 0.4 °C/min to yield the green body, a porous scaffold composed mainly of SiC particles.

PIP is a postprocessing procedure (Figure S3) where PCPs are infiltrated into the pores of the debinded sample to increase the density and structural integrity of the samples once pyrolyzed. Every cycle of PIP consists of three steps–vacuum infiltration with liquid PCP of the debinded structure, curing of the PCPs, and pyrolysis of the PCPs. A higher number of PIP cycles will lead to denser structures with improved mechanical properties, assuming that the porous structure remains open and can be infiltrated with liquid PCP. Durazane 1800 was chosen as the best PCP to fill the porous SiC particle scaffold due to two factors: (1) as a polysilazane, it will yield SiCN and SiOC, which can give excellent mechanical strength;^29,30^ and (2) the PCP itself is very low viscosity and flows into the porous SiC particulate scaffold easily, making the infiltration process faster and more complete.

The debinded samples were placed on the bottom of the round-bottom flask. Then, a vacuum pump was applied to lower the pressure below 5 kPa. After low pressure was achieved, Durazane 1800 was released dropwise (10 mL/min) onto the samples until they were fully immersed. The samples were left under vacuum for 20 min to equilibrate before removal from the flask, and another pyrolysis cycle was conducted.

Pyrolysis of the PDC-infiltrated sample turns the PCP into ceramic, obtaining a densified PDC composite. In a typical pyrolysis procedure, the sample was transferred into a tube furnace after polymer infiltration. The sample was heated to 170 °C from room temperature with a ramp rate of 1.2 °C/min to cure the PCP, and then the temperature was increased to 800 °C with a ramp rate of 0.48 °C/min. After dwelling at 800 °C for an hour, the sample was cooled to room temperature with a 1 °C/min cooling rate. During the entire pyrolysis process, argon flow (50 cm^3^/min, 99.95% ultrahigh purity) was used to maintain an inert environment in the tube furnace.

### 2.6 Characterization

Fourier-transform infrared spectroscopy (FTIR) was performed on a Bruker Vertex 70 IR spectrometer (Billerica, MA) equipped with a liquid nitrogen-cooled midband mercury cadmium telluride detector. Diamond attenuated total reflection (ATR) was used to analyze the resin and ceramic chemical compositions in the 500–2000 cm^–1^ range. The deconvolution of the FTIR peaks was performed using Gaussian fitting. For liquid samples, a thin layer was spread onto the crystal for analysis. For solid samples, a flat part of the sample was pressed down against the crystal. For powder samples, a sample press was used to press the powder against the diamond window to ensure good contact for analysis. The crystalline structure of the pyrolyzed samples was characterized by X-ray diffraction (XRD) (X-ray Diffractometer, Malvern Panalytical Empyrean, Malvern, United Kingdom) within a 2θ range of 30° to 75°. The microscopic morphology and elemental distribution of the samples were characterized by scanning electron microscopy (SEM) (Verios 5 XHR SEM, Waltham, MA) . The elemental composition of the samples was measured with X-ray photoelectron spectroscopy (XPS) (VersaProbe III, Chanhassen, MN) equipped with a monochromatic Al kα X-ray source (hν = 1,486.6 eV) and a concentric hemispherical analyzer.^31^ The samples were fractured in air immediately prior to introducing to the vacuum system to examine the cross-section and avoid minimizing any surface oxidation that was present. The density was determined using the Archimedes method. Since PIP does not change the dimensions of the sample,^32^ the linear shrinkage of the sample after debinding was calculated by the percent change in length after debinding, given by eq 1:
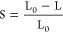
1Where S is the shrinkage of the sample after debinding, L_0_ and L stand for the length of the samples before and after debinding, respectively.

The flexural strength was measured using an MTS Criterion 43 (C43.504, Eden Prairie, MN) with an MTS 1kN S-beam load cell in a 3-point bend fixture. Flexural strength σ is given by eq 2 (ASTM C1341–13):
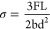
2where F is the fracture load, L is the support span length, b and d are the width and thickness of the sample, respectively.

Vickers microindentation (Qness Q60 A+, QATM, Austria) was employed on the polished cross sections of mounted bar samples. An applied load of 10 kgf (n = 5 per sample) was utilized to measure these ceramic materials’ hardness and fracture resistance. The indentation fracture toughness (K_IFR_) was calculated using the eq 3 given by Anstis et al.^33,34^ and the assumed elastic modulus of silicon carbide (Voigt-Reuss-Hill average, 434 GPa^33^). The crack lengths were measured using the average of five parallel lines (as described in Quinn’s method^35^) for horizontal and vertical diagonals with image analysis software (ImageJ, Figure S4).

Fracture toughness K_IFR_ is given by^33,34^
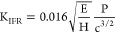
3where E and H is the elastic modulus and hardness of the sample, respectively, P is the peak load, and c is the crack length.

## 3 Results and Discussion

### 3.1 Fourier-Transform Infrared (FTIR) Spectroscopy

**Figure 4:**
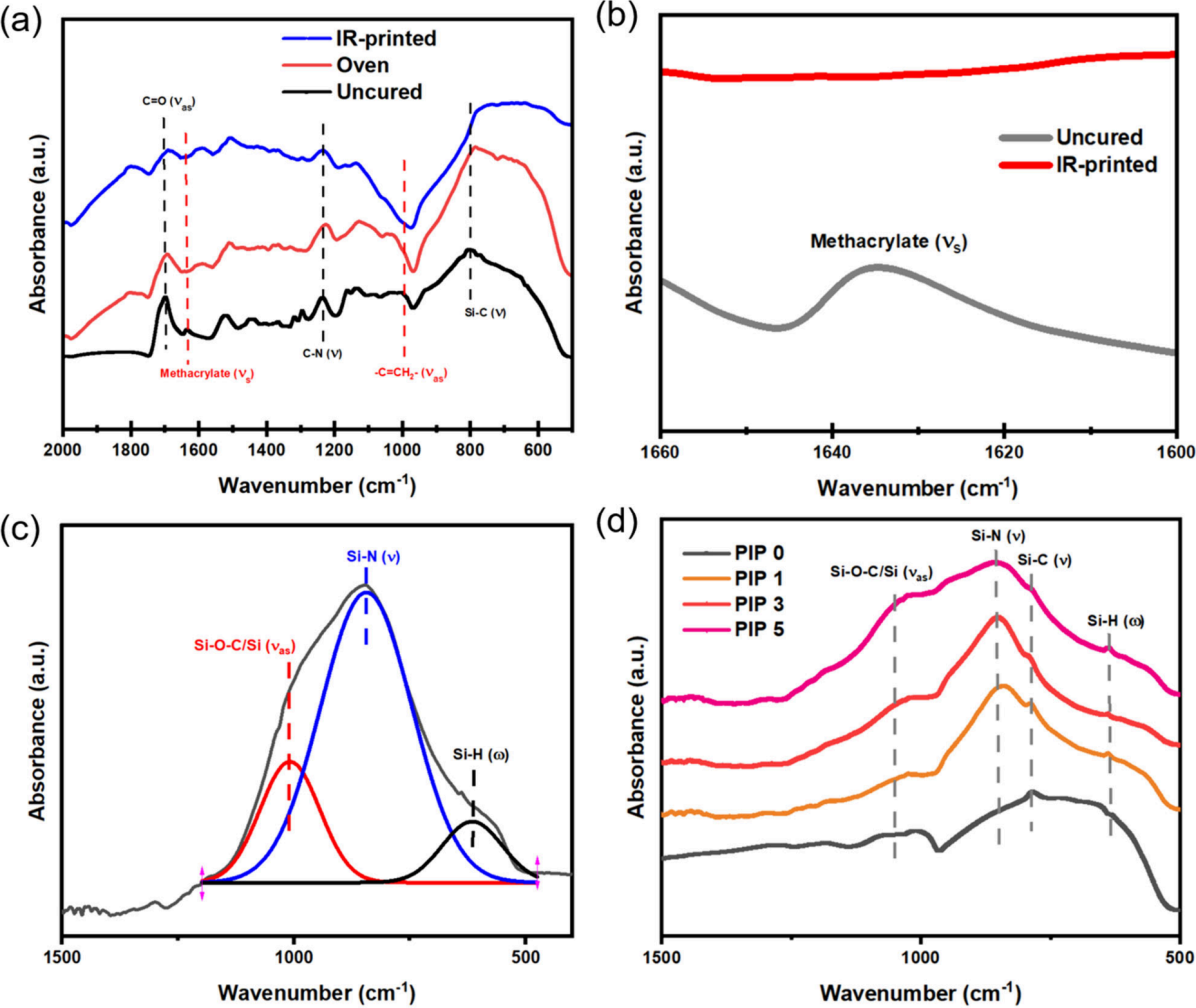
(a) FTIR spectrum of NIR-printed, oven-cured, and uncured *DUDMA-SiC 50:50* samples; (b) local FTIR spectrum of NIR-printed and uncured *DUDMA-SiC 50:50* samples; (c) deconvoluted FTIR spectrum of Durazane pyrolyzed at 800 °C; (d) FTIR spectrum of NIR-printed *DUDMA-SiC 50:50* samples after PIP.

FTIR analysis was conducted on uncured, oven-cured, and NIR-printed green body samples (Figure 4(a, b)) to compare the changes in the chemical composition of the resin between NIR printing and oven-curing methods. Additionally, the chemical composition of pyrolyzed Durazane PCP (Figure 4(c)) and debinded green body after PIP was also investigated (Figure 4(d)) with FTIR.

From Figures 4(a, b), it can be concluded that there are no significant differences in chemical composition between the oven-cured samples and NIR-printed samples in the mid-IR spectral region. The attenuation of the methacrylate symmetric stretching peak *ν*_*s*_ (C=C)^36^ at 1635 cm^–1^ and the disappearance of the asymmetric stretching band *ν*_*as*_ (−C–H) in the −C=CH_2_– moiety^36^ at around 994 cm^–1^ are evidence of acrylate curing after heat treatment. The FTIR spectrum in Figure 4(c) shows the chemical composition of Durazane after pyrolysis at 800 °C, which is identical to the PIP pyrolysis. The deconvoluted peaks at 1007 cm^–1^, 850 cm^–1^, and 620 cm^–1^ correspond to *ν*_*as*_ (Si–O–C/Si), ν (Si–N), and wagging band ω (Si–H),^37^ respectively. Figure 4(d) shows the FTIR spectra of PDC composite samples after PIP processing. This data shows the same ν (Si–O–C/Si), ν (Si–N), and ω(Si–H) peaks as the matrix PDC shown in Figure 4(c). Moreover, there is evidence of a weak *ν*_*as*_ (Si–O–Si)^38^ peak at 1090 cm^–1^, and a ν (Si–C) peak from 780–790 cm^–1^, indicating multiple ceramic components, including SiC, SiCN, SiO_2_, and SiOC in the final PDC composite.

### 3.2 X-ray Diffraction (XRD)

**Figure 5:**
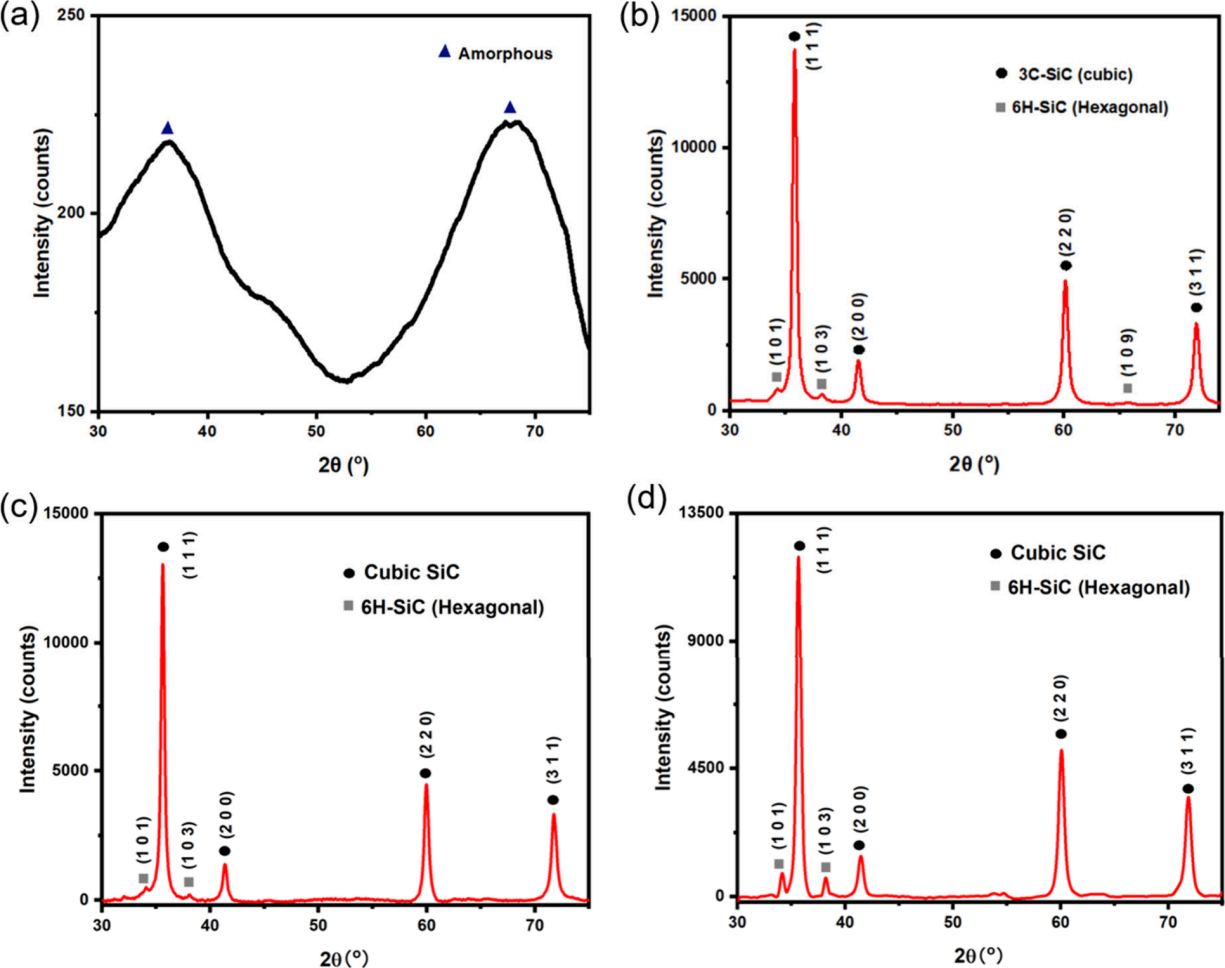
XRD patterns of: (a) pyrolyzed Durazane 1800; (b) SiC filler particles; (c) oven-cured sample after 3 cycles of PIP; (d) NIR-printed sample after 3 cycles of PIP.

The SiC particle fillers, matrix PDC, and the resulting PDC composites were analyzed with X-ray diffraction (XRD) to show the presence of crystalline phases in the materials (Figure 5). All pyrolyzed samples were treated under argon (50 cm^3^/min) at 800 °C with the same heating and cooling procedure detailed in the experimental section.

After pyrolysis of the PDC at 800 °C, two broad peaks at 2θ = 33.2° and 68.0° indicate there are only amorphous phases for pyrolyzed Durazane 1800, where the amorphous halo of these peaks demonstrates the material exists in cubic 3C-SiC phase.^39^ For oven-cured and NIR-printed samples after PIP, the peaks at 2θ = 35.8°, 41.5°, 60.1° and 71.9° correspond to crystalline cubic 3C-SiC particles; 2θ = 34.3°, 38.3°, 41.6°, 45.4°, 54.7°, 60.1°, 65.8°, 71.9° and 73.5° represent hexagonal 6H-SiC particles.^40^ Throughout each PIP cycle, XRD patterns of the NIR-printed and oven-cured samples showed they have the similar composition. Thus, only samples after 3 cycles of PIP are shown here. It can be concluded that the final ceramic part is made of highly crystalline SiC particles (mostly cubic phase) and amorphous PDC.

### 3.3 X-ray Photoelectron Spectroscopy (XPS)

**Figure 6:**
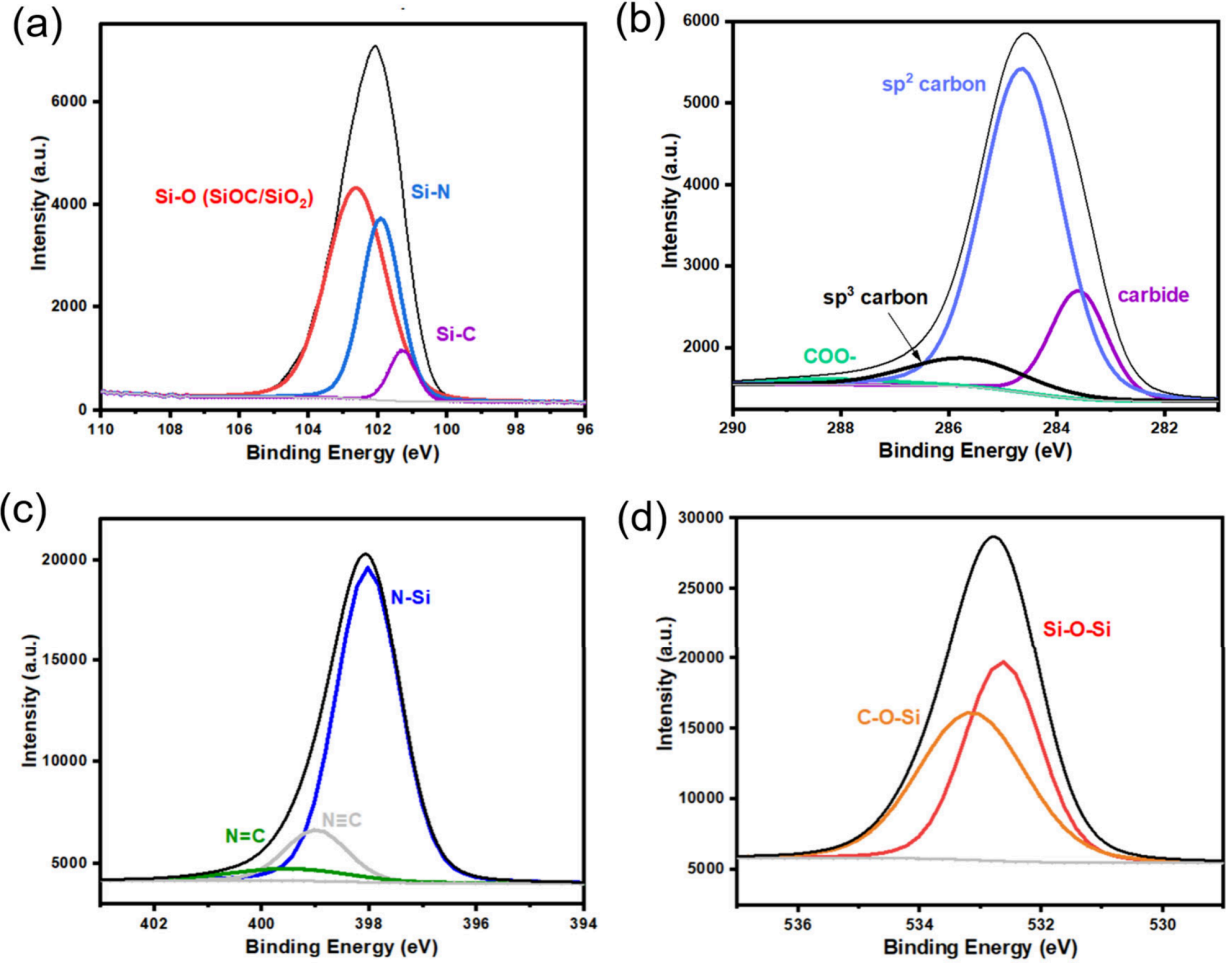
(a) XPS spectrum of Si 2p; (b) XPS spectrum of C 1s; (c) XPS spectrum of N 1s; (d) XPS spectrum of O 1s. All the spectra are taken for pyrolyzed Durazane.

X-ray photoelectron spectroscopy (XPS) experiments were conducted on thermally cured Durazane 1800 samples pyrolyzed under argon at 800 °C (Figure 6) to determine the matrix PDC composition in the presence of the SiC particles.

From the Si 2p XPS spectrum in Figure 6(a), peaks at 102.6, 101.9, and 101.3 eV correspond to Si–O (SiOC/SiO_2_), Si–N and Si–C, respectively. For the C 1s spectrum (Figure 6(b)), peaks at 288.1, 285.7, 284.6, and 283.6 eV correspond to COO–, sp^3^ carbon, sp^2^ carbon, and carbide, respectively. Turning to N 1s XPS spectra in Figure 6(c), the three peaks identified at 399.4, 399.0, and 398.0 eV are N=C, N≡C, and N–Si. Finally, the two peaks in the O 1s spectrum (Figure 6(d)) indicate two types of O: C–O–Si at 532.2 eV and Si–O–Si at 532.6 eV. From the XPS spectra, C, N, O, and Si are calculated to have 25.7 atom %, 18.8 atom %, 20.3 atom %, and 35.3 atom % abundance in the sample, respectively.

XPS spectra shows the elemental composition of the ceramic matrix of the PDC composite. When combining the results from XPS (Figure 6) and FTIR (Figure 4(c)), it can be concluded that the ceramic matrix from pyrolysis of Durazane is mainly made of a mixture of SiCN and SiOC, with some SiO_2_ in the sample. When considering the evidence from XRD (Figure 5(a)) and FTIR (Figure 4(d)), It can be concluded that the samples after PIP are mainly composed of crystalline 3C-SiC SiC particles (Figure 5(b-d)) with an amorphous SiCN and SiOC matrix.

### 3.4 Scanning Electron Microscopy (SEM)

**Figure 7:**
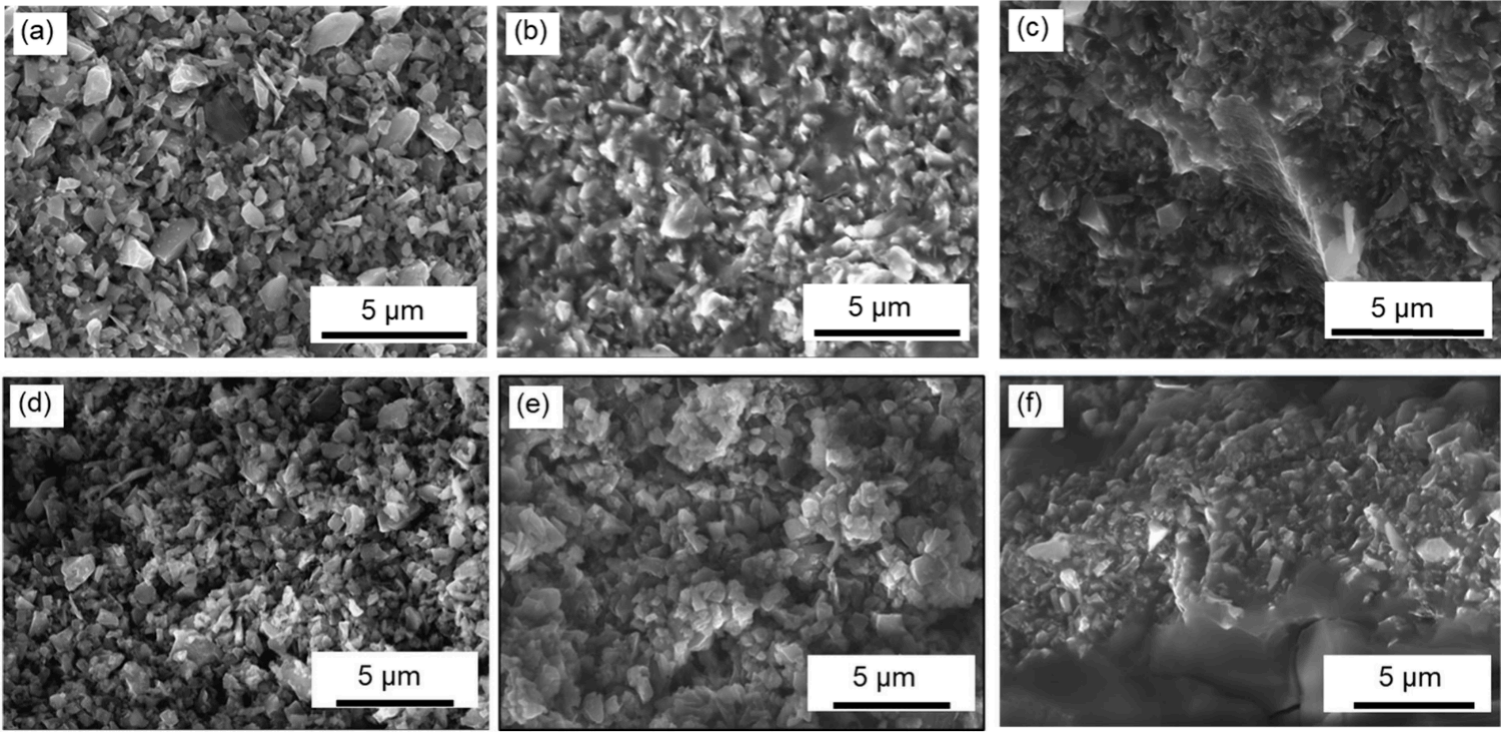
(a) Oven-cured sample after debinding (PIP 0); (b) oven-cured sample after 1 cycle of PIP (PIP 1); (c) oven-cured sample after 5 cycles of PIP (PIP 5); (d) NIR-printed sample after debinding (PIP 0); (e) NIR-printed sample after 1 cycle of PIP (PIP 1); (f) NIR-printed sample after 5 cycles of PIP (PIP 5). All images were taken on fractured surfaces.

Scanning electron microscopy (SEM) analysis was conducted to analyze the morphology of the samples and to determine how the PIP process changed the microstructure and porosity of the samples. The SEM micrograph of the NIR-printed samples and the oven-cured samples showed similar microstructures, Figure 7, where an increase in PIP cycles helped to decrease the gaps between SiC particles with increased PDC yield.

The *DUDMA-SiC 50:50* sample after debinding (PIP 0) showed a rough surface composed primarily of crystalline SiC particles due to the near-complete burnoff of the binder. There were no sample dimensional changes throughout the PIP cycles; the only dimensional change associated with the postprocessing procedure was the debinding of the green body, during which 9.4% linear shrinkage occurred following the removal of organic binders. Since the PIP process does not increase the volume of the samples, PCP infiltrated into the pores will turn into PDC within the porous body and densify the samples after pyrolysis. After one cycle of PIP, most of the SiC particles were held together with amorphous SiOC and SiCN from the PCP (Durazane) (Figure 7 (b)). However, pores are still visible in these samples, and the density after one PIP cycle was 1.76 g/cm^3^ for NIR-printed samples. After five cycles of PIP, almost all the particles in the sample appear to be consolidated, and there are no visible pores in the sample, according to microscopic analysis, Figure 7(f). Moreover, the samples after five cycles of PIP showed a nonporous structure, where fewer openings are present in the amorphous SiOC and SiCN matrix region.

**Figure 8:**
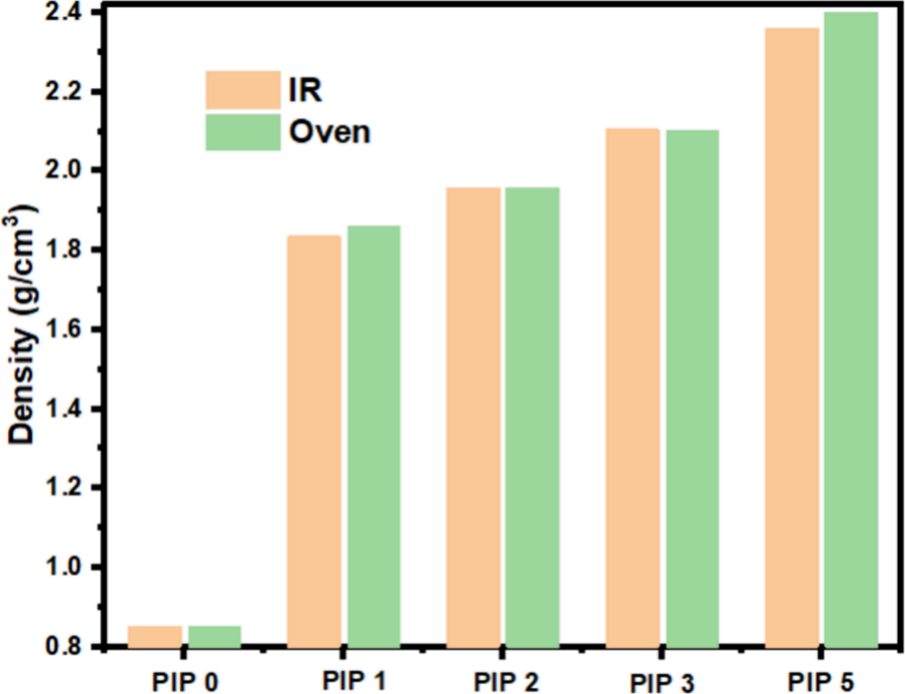
Density measurement of oven-cured and NIR-printed samples after different PIP cycles.

The density of the NIR-printed samples increased from 0.85 to 2.36 g/cm^3^ after five PIP cycles toward an estimated composite theoretical density of 2.60 g/cm^3^. The theoretical density was estimated assuming a composition of 40 wt % SiC (ρ = 3.21 g/cm^3^) and 60 wt % amorphous SiOC (ρ = 2.3 g/cm^3^). Figure 8 shows density measurements for NIR-printed and oven-cured samples as a function of the number of PIP cycles. This data demonstrates that the oven-cured and printed samples showed similar density increases during the PIP processes.

### 3.5 Mechanical Properties

As discussed in the previous section, PIP as a postprocessing method will densify and strengthen the porous debinded samples. Mechanical analyses, including three-point bending and microindentation, were performed to determine the flexural strength and fracture toughness of oven-cured and NIR laser-printed samples. This testing allowed us to assess the impact of PIP on the strength and fracture toughness of the samples.

**Figure 9:**
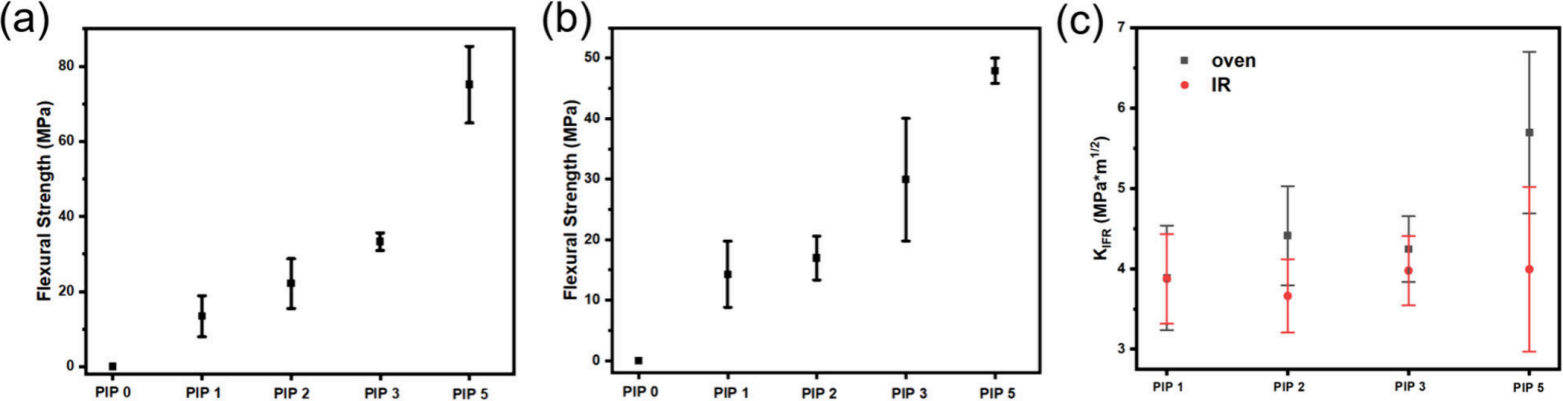
(a) Flexural strength of PIPed oven-cured *DUDMA-SiC 50:50* samples; (b) flexural strength of PIPed IR *DUDMA-SiC 50:50* samples; (c) fracture toughness of *DUDMA-SiC 50:50* samples. Error bars represent the standard deviation (±SD) of the measurement.

The debinded green body samples only have negligible strength (0.1 MPa). However, the flexural strength of oven-cured samples increased from 13.43 MPa (PIP 1) to 75.12 MPa (PIP 5) with a 459% increase (Figure 9(a)), which can be explained by the fact that the PDC composites were becoming denser with an increased PIP cycle count. The oven-cured and PIPed samples showed excellent flexural strength, 75.1 ± 10.2 MPa (PIP 5, Figure 9(a)), which is comparable to the 3D printed SiC PDC composite reported before with a flexural strength of 66.8 MPa.^41^ Compared to oven-cured samples, the NIR printed samples after PIP showed relatively lower mechanical strength, 47.9 ± 2.1 MPa (Figure 9(b)). This is likely due to the NIR-printed samples having more defects than oven-cured samples due to the layered nature of 3D printing. Additionally, the NIR-printed samples have a rougher surface finish due to the printer being only a first proof-of-concept. Typically, the flexural strength of polymer-derived ceramics (PDCs) is relatively low, leading researchers to enhance strength by adding fillers or whiskers. Our PDC composites show significantly improved strength compared to preceramic polymers alone and are comparable to reported values. For example, Cramer et al.^42^ 66.8 MPa using binder jet 3D printing with polycarbosilanes. Kemp et al.^10^ reported 56.4 ± 7.6 MPa by adding hBN in polysilazane. Xiong et al.^18^ obtained 33.2 MPa with SiC whiskers, and Huang et al.^43^ reported 15.8 MPa for porous SiC.

The indentation fracture toughness increased by 47% for oven-cured samples after 5 PIP cycles, while it only increased negligibly (3.1%) for NIR-printed samples, where it reached 5.7 ± 0.9 MPa·m^1/2^ for oven-cured samples and 4.0 ± 0.9 MPa·m^1/2^ for NIR-printed samples (Figure 9(c)). Higher PIP cycles tend to toughen the microstructure and enhance mechanical properties as they increase the fracture resistance of the samples (Table S1). After a PIP cycle, the infiltration PDCs are softer and act as plastic regions to absorb more energy during fracture (Figure S4 and Table S1). However, IR-printed samples exhibit a less pronounced improvement in fracture toughness. This can be attributed to the presence of inherent defects introduced during the IR printing process, as evidenced in the SEM micrograph. These defects likely act as stress concentrators and preferential sites for crack initiation, limiting the efficacy of the toughening mechanisms provided by the plastic matrix. The average reported fracture toughness of Si–C–N and Si–O–C systems is 0.56–3 MPa·m^1/2^.^44^ Thus, the PDC composite in this report shows an improvement over materials systems that have been demonstrated previously.

## 4 Conclusions

This work presents the development of a novel thermal SLA printer to fabricate PDC composite parts, solving issues with UV-based SLA printing of PCPs. Additionally, a thermal printing resin composition was reported in this study, with a SiC particle loading of 50 wt %. FTIR analysis showed small compositional difference between laser-cured and oven-cured PDC samples, which supports the feasibility of NIR thermal printing to obtain PDC materials with good properties. The crystalline structure of the particles and the matrix was characterized by XRD analysis, where the results confirm that the filler particles are crystalline 3C-SiC particles while the matrix is amorphous. The chemical composition of the PDC composite after PIP was measured with FTIR and XPS, demonstrating that amorphous SiOC and SiCN are the main components of the matrix. Following PIP, the printed parts demonstrated high mechanical strengths (48 MPa) and fracture toughness (4.0 ± 0.9 MPa·m^1/2^). SEM images showed how the PIP procedure strengthened the samples–consolidating the particulate filler with an amorphous PDC matrix from PCP pyrolysis. During printing, heat conduction in the resin causes thermal energy to diffuse beyond the irradiated area, reducing resolution. Future work will focus on improving heat dissipation through enhanced convection and conduction cooling, which could mitigate thermal diffusion and improve resolution. This prototype demonstrates NIR laser stereolithography for preceramic polymers, addressing challenges in UV-based printing and showing the potential of thermal SLA for fabricating PDC 3D-printed parts.

## Supporting Information Available

Video of NIR thermal printing process (MP4)
Laser and printer parameters, PIP experimental setup, microindentation images and summary of indentation mechanical properties, energy dispersive spectroscopy of samples after PIP, FTIR spectra of silicon carbide particles before and after debinding in air, XPS survey spectrum of pyrolyzed Durazane (matrix PDC) (PDF)
