# Supplementary material for: Thermal Stereolithography of SiC-Loaded Acrylate Resins with Polymer-Derived Ceramic Infiltration
Source: ACS Appl Eng Mater. 2025 Apr 1;3(4):947–56. doi: 10.1021/acsaenm.5c00054 (PMC12038839; doi:10.1021/acsaenm.5c00054)
Supplement: Supplementary file 2 — em5c00054_si_002.pdf [file em5c00054_si_002.pdf]

Supporting Information:

Thermal Stereolithography of SiC-Loaded Acrylate Resins with Polymer-Derived Ceramic Infiltration

*Evelyn Wang[b], Shruti Gupta[a], Joseph Fortenbaugh[a,d], Caillin J. Ryan[a], Christopher M. DeSalle[a], Jeffrey R. Shallenberger[c], Douglas E. Wolfe[a], Benjamin J. Lear[e], Michael A. Hickner[b,]**

* Corresponding Author: Michael A. Hickner, Department of Chemical Engineering and Materials Science, Michigan State University, 428 S Shaw Ln, Rm 2258, East Lansing, MI, 48824. E-mail: mhickner@msu.edu

AUTHOR ADDRESS

a: Department of Material Science and Engineering, Pennsylvania State University, University Park, Pennsylvania, 16801, United States.

b: Department of Chemical Engineering and Materials Science, Michigan State University, East Lansing, Michigan, 48824, United States.

c: Materials Research Institute, The Pennsylvania State University, University Park, Pennsylvania 16802, United States.

d: Oak Ridge National Laboratory, Oak Ridge, Tennessee, 37830, United States.

e: Department of Chemistry, Pennsylvania State University, University Park, Pennsylvania, 16801, United States.

Laser and printer parameters

The energy density of the laser is given by equation (S1):

$$\eta = \frac{P}{D \cdot v} \tag{S1}$$

where η is the energy density of the laser beam, P is the power output of the laser, D is the beam's diameter, and ν represents the laser beam's scanning speed on the surface of the printing resin.

The laser output was 2.96 W, the diameter of the laser beam was 2.0 mm, and the scanning speed was 5 mm/s. The calculated energy density of the laser beam was 29.6 J/cm^2.

Laser parameters and setup

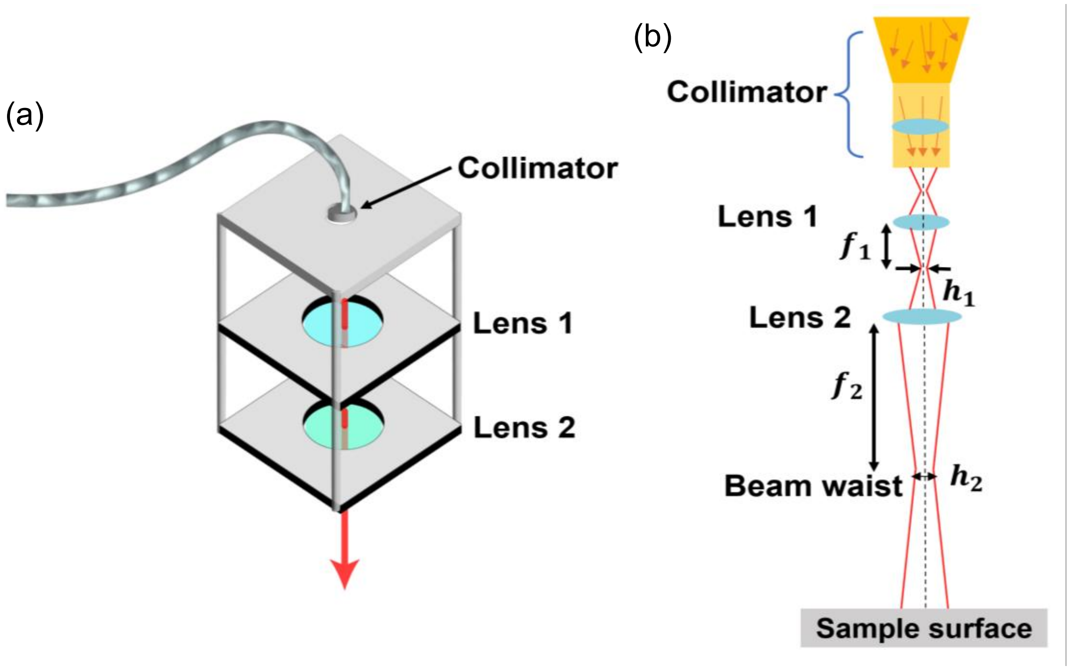

Figure S1. (a) Configuration of the optical cage system for NIR thermal SLA printer; (b) diagram of optical beam path through the cage system.

The configuration of the optical cage system for the NIR thermal SLA printer is depicted in Figure S1, where three components make up the system – a laser collimator and two lenses. The laser generated from the diode will be randomly scattered in the fiber optic; therefore, a collimator and lenses are needed to collimate and control the beam divergence. The beam expander will change the beam waist diameter (h) and divergence angle. Since a larger beam waist will lead to lower divergence, a set of beam expanders was used to ensure printing accuracy (Figure S1). When placing two lenses apart at a distance equal to the sum of their focal lengths, we have:

$$\frac{f_1}{f_2} = \frac{h_1}{h_2} \quad \text{(S2)}$$

where f_1 and f_2 are the focal lengths of two lenses, respectively, and h_1 and h_2 are the radius of the beam entering and leaving the two lenses, respectively. The beam parameters: h_1 = 0.8 mm, h_2 = 3 mm, f_1 = 4 mm, and f_2 = 15 mm were used to maintain high printing resolution.

Images of NIR-printed samples after post-processing

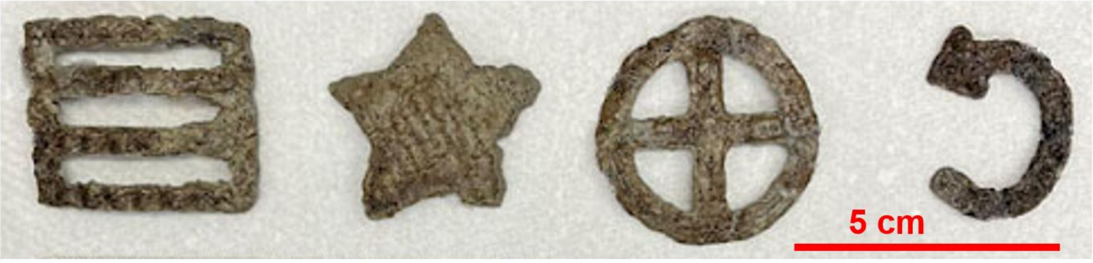

Figure S2. NIR-printed samples after debinding and 5 cycles of PIP.

Post-processing – polymer infiltration and pyrolysis (PIP) experimental setup

The experimental setup for PIP processing is shown in Figure S3, which consists of four parts – a dropper funnel, a vacuum pump, a manometer, and a three-necked flask with a sealable middle opening.

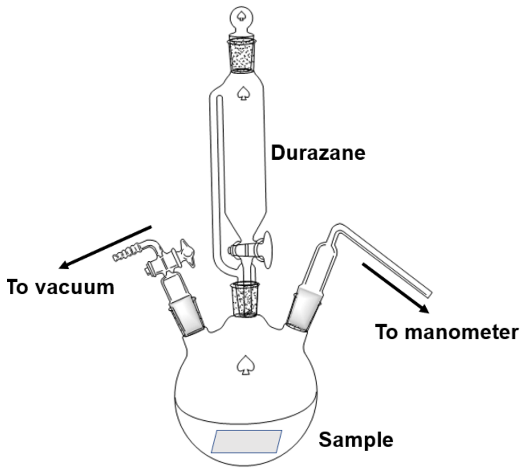

Figure S3. Polymer infiltration pyrolysis (PIP) reactor.

Micro-indentation fracture toughness

The fracture toughness of the samples was measured using the indentation method described in the main manuscript. The parameters that need to be measured for this method are hardness, crack length, and the force applied to the tip. These results are shown in Figure S4 and Table S1.

Figure S4 provides images of the post-indentation microstructures using optical microscopy. There are some differences in the underlying microstructure between the oven/IR-cured samples. In Figure S4 (a - d), a less homogeneous microstructure is observed with the oven-curing process, while Figure S4 (e – h) shows a more uniform microstructure with the IR-curing process. Nevertheless, in both heat treatments, notably smaller indentation crack lengths are apparent in the samples with the least PIP cycles (Figure S4 (a, e)) compared with the

greatest PIP cycles (Figure S4 (d, h)). This corroborates the mechanistic strengthening effects of the flexure testing results seen with cumulative PIP cycles, where the more plastic PDC matrix regions absorb energy and lengthen the crack paths.

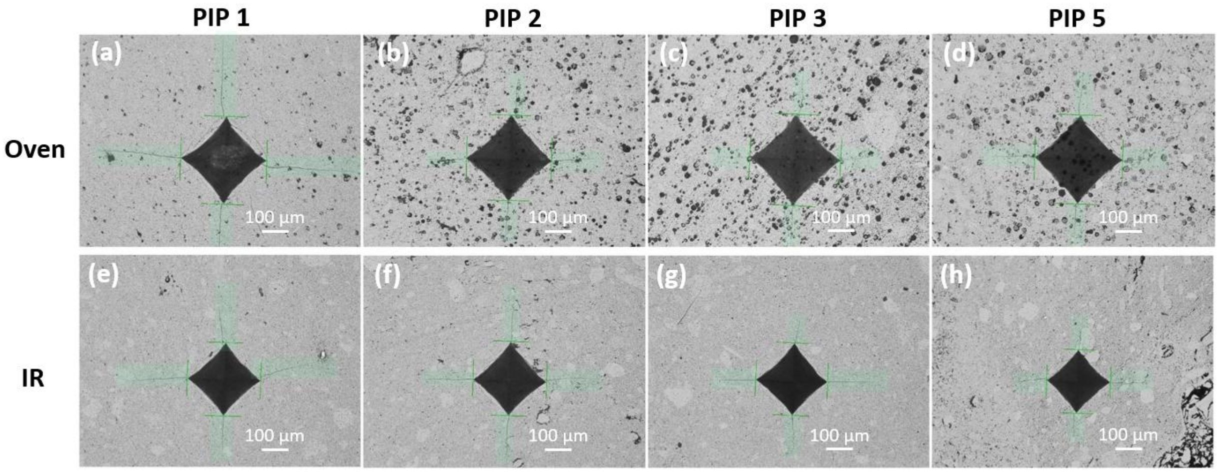

Figure S4. Indentation on the samples, crack lengths, and horizontal and vertical diagonals are marked. (a-d) oven-cured samples after 1, 2, 3, 5 cycles of PIP; (e-h) IR-printed samples after 1, 2, 3, 5 cycles of PIP.

Table S1 shows the effects of PIP cycles on the indentation mechanical properties. In the third column (Vickers hardness, HV_{10}), both the oven/IR-curing treatments with the highest number of PIP cycles demonstrated substantially higher hardness than that of the lower PIP cycle samples. Furthermore, the IR curing treatments showed systematically higher hardness (~ 42 %) than oven-cured samples under identical PIP cycles. This trend is also observed in the fourth column (crack-to-diagonal length ratio, c/a), where the c/a ratio shows a similar systematic increase between the IR and oven-cured samples. Additionally, the fracture resistance in the fifth

column (indentation fracture resistance, F/c) shows a correlated monotonic increase with the hardness in the third column, reinforcing that greater PIP cycles tend to strengthen the microstructure and provide enhanced mechanical properties. This is further reflected in the estimated indentation fracture toughness values in the last column (K_{IFR}), which shows a greater toughness for the oven-cured sample than the IR-cured samples. This may be attributed to greater available toughening mechanisms with more plastic PDC matrix, as the lower resistance to plastic deformation dominates the overall irreversible dissipation of energy from the applied mechanical load. Consequently, the increased plasticity results in less crack propagation under identical loads between the IR/oven-cured samples.

Table S1. Tabulated summary of indentation mechanical properties across PIP cycle and heat treatment types. Values are averages bounded by ± 95 % confidence intervals; parenthesis indicates min-max measured values.

Type	PIP Cycle	HV_{10} (kg/mm^2)	c/a	F/c (N/mm)	K_{IFR} (MPa·m$^{1/2}$)
Oven	1	189 ± 16 (168-214)	2.18 ± 0.25 (1.94-2.63)	290 ± 29 (239-324)	3.89 ± 0.57 (2.88-4.44)
	2	205 ± 10 (190-219)	2.02 ± 0.18 (1.79-2.34)	325 ± 26 (278-351)	4.41 ± 0.54 (3.49-5.14)
	3	189 ± 17 (174-215)	2.03 ± 0.11 (1.85-2.18)	308 ± 22 (277-330)	4.25 ± 0.36 (3.75-4.79)
	5	241 ± 49 (187-314)	1.75 ± 0.16 (1.44-1.92)	405 ± 51 (324-465)	5.70 ± 0.88 (4.60-7.33)
	Pooled	206 ± 16 (168-314)	2.00 ± 0.11 (1.44-2.63)	332 ± 25 (239-465)	4.56 ± 0.42 (2.88-7.33)
IR	1	274 ± 6.8 (268-287)	2.31 ± 0.18 (2.07-2.50)	328 ± 29 (298-367)	3.87 ± 0.49 (3.38-4.49)

	2	276 ± 12 (262-295)	2.40 ± 0.20 (2.23-2.80)	317 ± 24 (269-335)	3.66 ± 0.40 (2.87-4.01)
	3	272 ± 25 (223-291)	2.26 ± 0.17 (2.02-2.50)	333 ± 16 (308-359)	3.98 ± 0.38 (3.43-4.45)
	5	348 ± 46 (262-404)	2.40 ± 0.37 (1.99-2.99)	361 ± 57 (298-433)	3.99 ± 0.90 (2.87-5.13)
	Pooled	293 ± 19 (223-404)	2.35 ± 0.12 (1.99-2.99)	335 ± 18 (269-433)	3.88 ± 0.27 (2.87-5.13)
